# Herbicide Metolachlor Causes Changes in Reproductive Endocrinology of Male Wistar Rats

**DOI:** 10.5402/2012/130846

**Published:** 2012-04-18

**Authors:** Francielle Tatiane Mathias, Renata Marino Romano, Hanan Kaled Sleiman, Claudio Alvarenga de Oliveira, Marco Aurelio Romano

**Affiliations:** ^1^Department of Pharmacy, State University of Centro-Oeste, R. Simeao Camargo Varela de Sa, 03, 85040-080 Guarapuava, PR, Brazil; ^2^Department of Animal Reproduction, Faculty of Veterinary Medicine, University of Sao Paulo, Avenida Prof. Dr. Orlando Marques de Paiva, 87, 05508-270 Sao Paulo, SP, Brazil

## Abstract

S-metolachlor is a chloroacetanilide herbicide widely used in the agriculture to control weeds and was demonstrated that it increases the activity of the aromatase enzyme in cell cultures, which may culminate as endocrine disruption action *in vivo*. To investigate this hypothesis, prepubertal Wistar male rats were exposed to metolachlor (5 or 50 mg/kg/day, NOEL for reproductive toxicity: 23.5–26.0 mg/kg/day) from PND23 (postnatal day) to PND53. During this period, the growth of the animals and the age and weight at puberty were recorded. In PND53, tissues were collected and the analysis of LH, FSH, testosterone, dihydrotestosterone (DHT), estradiol serum concentrations, morphometric evaluation of the seminiferous epithelium, and weight of the testes and the seminal vesicle (undrained and drained) was performed (Statistical difference: *P* < 0.05). Metolachlor caused an increase in serum concentrations of testosterone, estradiol, and FSH and a reduction in DHT but did not alter the LH. There were also observed a higher amount of fluid in the seminal vesicles, precocious puberty, and changes in morphology of the seminiferous epithelium of treated animals. We demonstrated in this paper that prepubertal exposure to S-metolachlor caused changes in reproductive endocrinology of male rats.

## 1. Introduction

The endocrine system may be the main target for the toxic manifestation of pesticides and may result in reproductive alterations, especially in steroid-hormone-dependent functions [1, 2]. Endocrine disruptors were defined by Kavlock et al. [3] as exogenous agents that interfere with the production, release, transport, metabolism, binding, action, or elimination of natural hormones responsible for the maintenance of homeostasis and the regulation of developmental processes. These disturbances may potentially cause risk to population by impairing their capacity to reproduce [4], being that occupational exposure to pesticides has been linked to a reduction in the quality of semen and a greater rate of infertility and miscarriage [5, 6].

S-metolachlor [S-2-chloro-N-(2-ethyl-6-methylphenyl)-N-(2-methoxy-1-methylethyl) acetamide] is a chloroacetanilide herbicide widely used in the agricultural sector to control weeds in corn, cotton, and soybean plantations, among other crops. The commercial formula of S-metolachlor comprises primarily of 88% S-enantiomer and 12% R-enantiomer, although only the S-enantiomer is biologically active. Compared to metolachlor (50% S-enantiomer and 50% R-enantiomer), S-metolachlor represents an important reduction in risk to users, consumers, and the environment [7, 8] since formulations of S-metolachlor in proportions above 80% have higher herbicide activity and a smaller amount of product may be used [9].

After agricultural application, metolachlor has been observed to persist longer in the subsoil than on its surface. Furthermore, great quantities of bonded residues and degradation products have been observed on the soil surface as a result of the increase in absorption of the soil and the biodegradation of metolachlor, because there is more organic material in this layer. However, this mechanism becomes saturated and metolachlor forms bond residues in the soil and is exposed to solar action or it may contaminate ground water [10, 11], or it may even become volatile in the environment [12].

Human exposure to chemical contaminants is practically inevitable, and the consequences to human health depend on the levels of environmental exposure. It has been recognized that environmental contaminants are capable of bonding with gonadal steroid receptors, which may imitate the action of steroid hormones and alter their production output. The detection of contaminant residues in human serum, follicular liquid, and seminal plasma [13, 14], together with reports of a supposed drop in semen quality [15–17], has highlighted the concern that exposure to environmental pollution can affect human fertility.

Adverse reproductive effects attributed to pesticides, including their effect on fertility, have also been well established in *in vivo* and *in vitro* studies [18–22]. One important observation was that metolachlor increases the activity of the aromatase enzyme in JEG-3 cell cultures [23]. This enzyme is responsible for the conversion of testosterone into estradiol, and the increase in it activity might result in alteration of the amount of testosterone, estradiol, and di-hydrotestosterone (DHT), since it is also produced from conversion of testosterone. It makes necessary an investigation into whether metolachlor may alter the production of sexual hormones *in vivo* and cause disturbances in the reproductive male development.

Based on these evidences and on the interest to determine the toxic effects of metolachlor on the male reproductive endocrinology, we used prepubertal rats as an experimental model, in an experimental design previously described [20]. After weaning, the exposure to metolachlor was initiated and the growth of the animals and the age and weight at puberty was evaluated. At the end of the period of exposure, blood samples of male rats of 53 days old were collected and subjected to analysis of LH, FSH, testosterone, dihydrotestosterone (DHT), and estradiol serum concentrations. The testes were submitted to morphometric evaluation of the seminiferous epithelium, through the analysis of histological sections. The weight of the testes and the seminal vesicle (undrained and drained) were also recorded to evaluate the effect of the treatment on the reproductive development.

## 2. Material and Methods

### 2.1. Chemicals

The product used was a commercial formulation of Dual Gold (Syngenta AG; Syngenta Protecao de Cultivos Ltda., São Paulo, Brazil), with an S-metolachlor base; this formulation contained 960 g/L (96%) of S-metolachlor formulated as an emulsifiable concentrate.

### 2.2. Animals, Experimental Design, and Treatment

Thirty newly weaned male Wistar rats were used, born of females that were monitored from the 17th day of pregnancy, in order to determine their offspring's exact days of birth. On the fourth postnatal day (PND4), the litters were culled to eight pups per female and maintained thus until weaning (PND21). The rats were kept in a 12:12 hour dark/light photoperiod cycle, at a controlled room temperature (23 ± 1°C), and they were fed with a commercial balanced ration mixture for rats and given water *ad libitum*. The animals were subjected to the experimental treatment regime from PND23 to PND53. The metolachlor was diluted in a watery suspension and administered once a day, *per os* (gavage) at a volume of 0.25 mL/100 g of body weight, between 7 and 8 AM. The toxicological analysis of metolachlor determined the following parameters that we consider relevant for the choice of doses used in this study: DL50 oral toxicity of metolachlor for rats (2780 mg/kg), maternal and developmental toxicity NOELs (300 mg/kg/day), and reproductive NOEL (23.5–26.0 mg/kg/day) United States Environmental Protection Agency [24]. Therefore, we used the dose of 5 mg/kg/day (MT5) and 50 mg/kg/day (MT50). The control group was treated in the same manner, but with deionized water instead of metolachlor. Each group comprised of ten animals. All procedures were carried out in accordance with Brazilian College of Animal Experimentation standards.

### 2.3. Preputial Separation (PPS)

The PPS is a marker of puberty in male rat and is used in reproductive toxicological protocols [25]. It is a cornifying process that leads to cleavage of the epithelium forming the stratified squamous lining of the prepuce of the penis and is a sign of puberty. This process is an essential prerequisite for acquisition of complete ejaculation [26] and is dependent of androgen [27]. This method was undertaken from PND33 and was carried out once a day at the time of balanopreputial separation, by means of gentle tissue manipulation. During this period, the animals were also weighed.

### 2.4. Reproductive Organ Weights

In order to evaluate the effect of metolachlor on the reproductive organs development, the testes and seminal vesicle were weighted and the absolute values were transformed into relative weights as mg/100 g of body weight. The seminal vesicle was weighted with fluid (undrained) and after fluid removal (drained).

### 2.5. Histology and Morphometry of Seminiferous Epithelium

The testes were fixed in Bouin's solution for 8 hours, treated with alcohol, embedded in paraffin, and then cuts of 5 *μ*m were prepared as stained slides with hematoxylin and eosin as previous described [20]. Briefly, the slides were observed initially under 40× magnification, in order to ascertain the general organ architecture. Next, 100× magnification was used for a more detailed analysis of the architecture of the seminiferous tubules. This included analyzing the linear morphometry of the seminiferous tubules by determining the tubular diameter (measured from the basal lamina to the basal lamina in the opposite direction), the thickness of the seminiferous epithelium (from the basal lamina to the neck of the elongated spermatids), and luminal diameter. Ten fields per cut per animal were selected within the histological cuts, in the transverse direction of the tubules. For each tubule, the averages were calculated for the measurements indicated and, then, the average of each field was also calculated. The measurements for each animal were obtained through the measurements of all the analyzed fields.

### 2.6. Hormone Measurements

Blood was collected via cardiac puncture in 53-day-old animals between 07:30 and 08:30 AM. and was centrifuged and subjected to serum levels of total testosterone and estradiol by radioimmunoassay, using commercial kits (Testosterone Total MPBiomedicals; Estradiol Coat-A-Count, Siemens Health Care Diagnostics, Los Angeles, CA, USA), Luminex xMAP technology for rat LH and FSH from Millipore Corp. (Milliplex MAP rat pituitary panel, Billerica, MA, USA), and ELISA kit for rat dihydrotestosterone DHT (Uscn Life Science Inc, NC, USA). All methods were performed according to manufacturer instructions. The minimum sensitivity was 0.9 ng/dL for testosterone, 1.57 pg/mL for estradiol, 2.91 pg/mL for LH, 31 pg/mL for FSH, and 13.25 pg/mL for DHT. The intrassay coefficient was <5% for testosterone, <3% for estradiol, <2.5% for LH, <4.7% for FSH, and <3% for DHT.

### 2.7. Statistical Analysis

The data were first submitted to Kolmogorov-Smirnov tests for normality and the Bartlett test for homoscedasticity. The analysis of body growth was performed using the multiway analysis of covariance for repeated measures using the weight at weaning as a cofactor (MANCOVA) by a general linear model (GLM). The weights were compared among different groups and different ages, considering the expected changes with age. The day of PPS was compared using nonparametric analyses with the Kruskal-Wallis test followed by the *post hoc* Dun test. The weights of seminal vesicle (drained and undrained) were compared by two-way ANOVA followed by the Tukey HSD *post hoc* test. All other parameters were analyzed by one-way ANOVA followed by the Tukey HSD *post hoc* test. All analyses were performed with Statistica 7.0 (Statsoft Inc, Tulsa, OK, USA). Statistical differences were considered significant when the value of *P* < 0.05. The values were expressed as means and the standard error of the mean (± SEM) for parametric and interquartile ranges of nonparametric analysis.

## 3. Results

### 3.1. Prepubertal Exposure to Metolachlor Alter the Serum Concentrations of Reproductive Hormones

**Figure 1:**
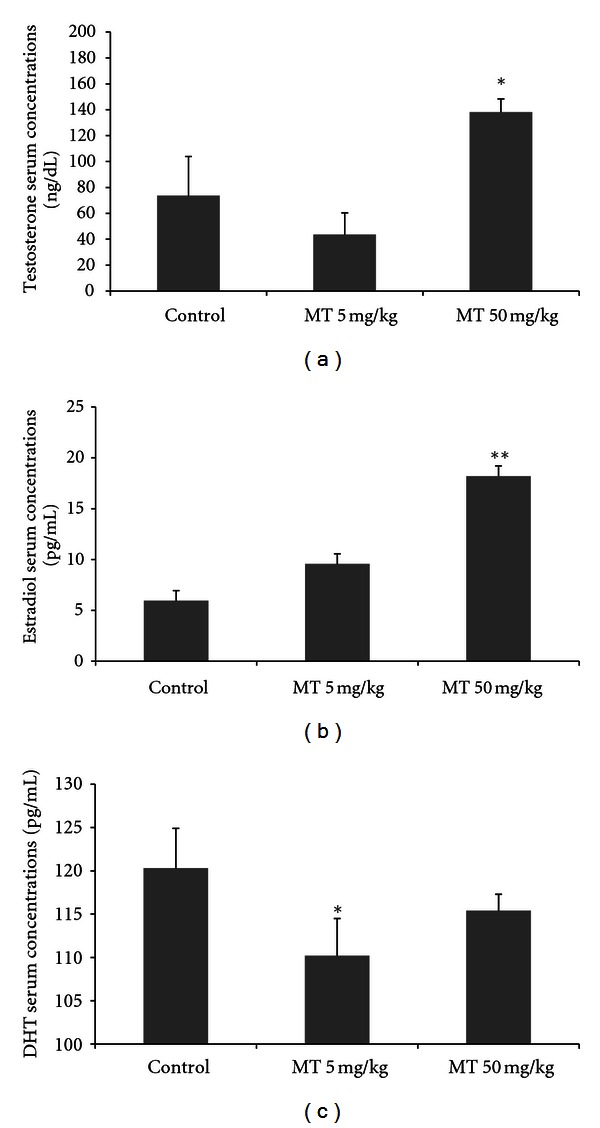
Serum concentrations of testosterone (ng/dL) (a), estradiol (pg/mL) (b), and dihydrotestosterone (pg/mL) (c) at 53 days old in male rats treated with the herbicide metolachlor during the prepubertal period. Doses: control = 0 mg/kg, MT5 = 5 mg/kg and MT50 = 50 mg/kg. Results are expressed as mean ± SEM, ANOVA, *n* = 10 animals/group, *P < 0.05, **P < 0.01.

**Figure 2:**
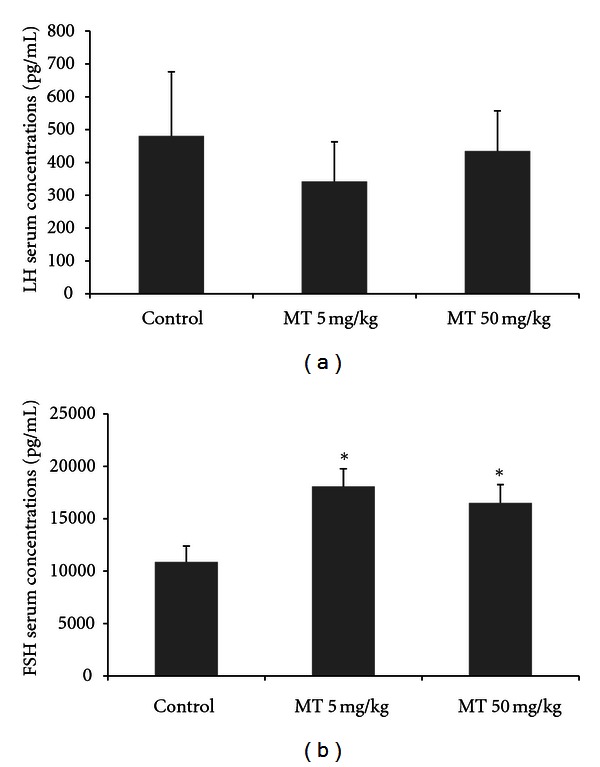
Serum concentrations of LH (pg/dL) (a) and FSH (pg/mL) (b) at 53 days old in male rats treated with the herbicide metolachlor during the prepubertal period. Doses: control = 0 mg/kg, MT5 = 5 mg/kg and MT50 = 50 mg/kg. Results are expressed as mean ± SEM, ANOVA, *n* = 10 animals/group, *P < 0.05.

The effect of daily exposure to the herbicide on the reproductive endocrinology was assessed by determining the serum concentrations of testosterone, estradiol, DHT, LH, and FSH in blood samples from 53-day-old rats. The serum concentration of testosterone was significantly different between the control and treated groups, increasing around 88% in MT50, as compared to the control and also with regards to MT5 (one-way ANOVA, *P* < 0.01, Figure 1(a)). The serum concentrations of estradiol were higher in the MT50 group, at around 300%, compared to control (one-way ANOVA, *P* < 0.01, Figure 1(b)). The serum concentrations of DHT were slightly reduced in MT5 (one-way ANOVA, *P* < 0.05, Figure 1(c)), but is not altered in MT50. The LH serum concentrations were not altered by the treatment (one-way ANOVA, *P* < 0.05, Figure 2(a)), but FSH was elevated in both treated groups, MT5 and MT50 (one-way ANOVA, *P* < 0.05, Figure 2(b)).

### 3.2. Prepubertal Exposure to Metolachlor Causes Changes in the Seminiferous Architecture

**Figure 3:**
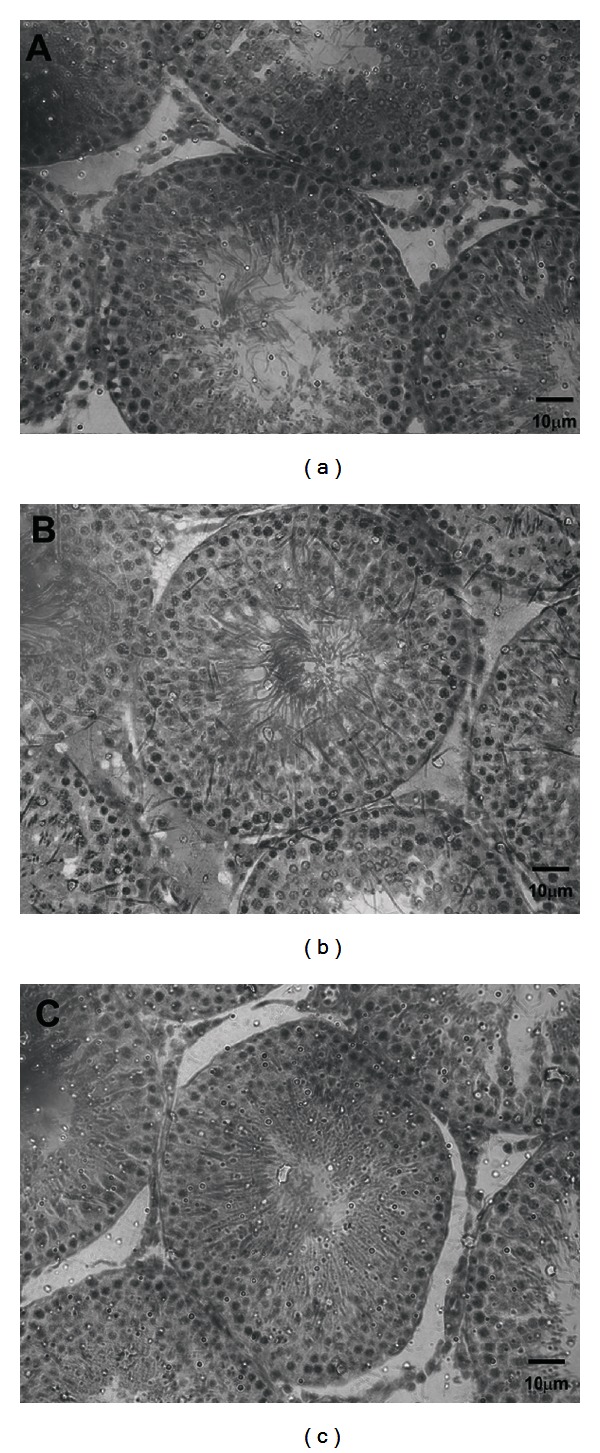
Effects of the herbicide metolachlor on testicular morphology of the control group (a) and treated groups at the doses of 5 mg/kg-MT 5 (b) and 50 mg/kg-MT 50 (c). The seminiferous tubules of treated groups (b, c) presented reduction in luminal diameter (LD) and greater epithelial height in relation to control group (a). *Scale bar *= 10 *μ*m. Hematoxylin and eosin stain.

The morphometry of the seminiferous epithelium may be related to spermatogenesis process. MT5 group presented larger epithelial height and reduced luminal diameter, with no alterations in the tubular diameter. MT50 group also presented increase in the epithelial height and was verified increase in the tubular diameter (one-way ANOVA, Table 1, Figure 3).

### 3.3. Metolachlor Increases the Amount of Seminal Vesicle Fluid

**Figure 4:**
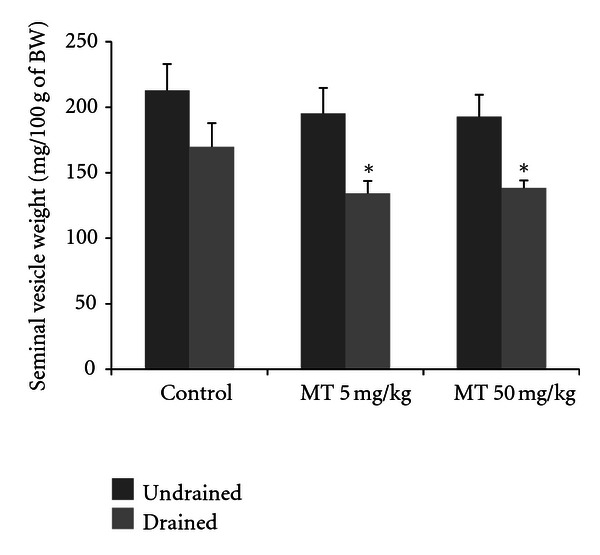
Seminal vesicle undrained (a) and drained (b) weights at 53 days old in male rats treated with the herbicide metolachlor during the prepubertal period. Doses: control = 0 mg/kg, MT5 = 5 mg/kg and MT50 = 50 mg/kg. Results are expressed as mean ± SEM, two-way ANOVA, *n* = 10 animals/group, *P < 0.05.

The seminal vesicles were also weighed to ascertain the effect of metolachlor. No significant differences were observed for the weight of seminal vesicles with fluid content (undrained). However, after the drainage of the seminal fluid (drained), the MT5 and MT50 groups presented a greater volume of seminal fluid than the control group (two-way ANOVA, *P* < 0.05, Figure 4).

The testes were weighted to ascertain possible alterations caused by daily exposure to metolachlor, and this treatment did not significantly alter the relative testicular weight of the animals among the treated groups (one-way ANOVA, *P* > 0.05).

### 3.4. Prepubertal Exposure to Metolachlor Anticipates the Onset of Puberty

**Figure 5:**
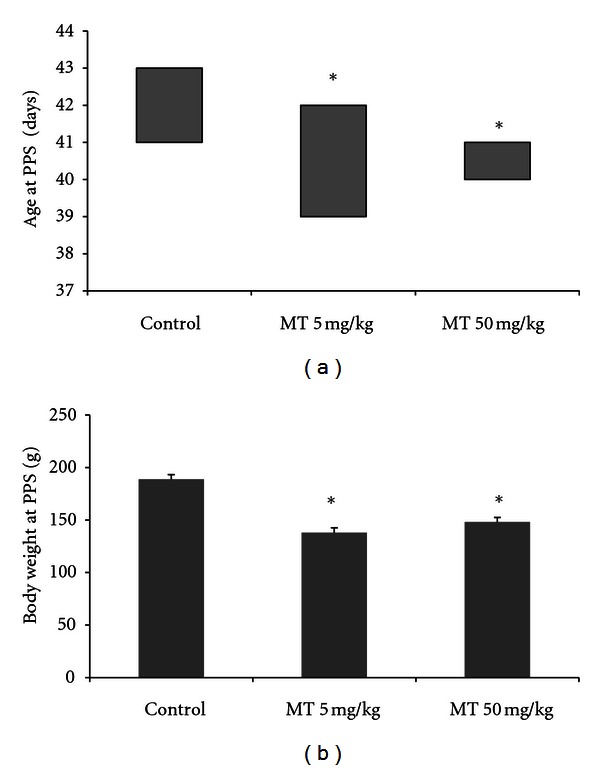
Age (a) and body weight at PPS (b) in male rats treated with the herbicide metolachlor during the prepubertal period. Doses: control = 0 mg/kg, MT5 = 5 mg/kg, and MT50 = 50 mg/kg. Results are expressed as median and interquartile ranges (a) and mean ± SEM (b). Kruskall-Wallis (a) and ANOVA (b), *n* = 10 animals/group, *P < 0.05.

The separation of preputial mucosa and exposure of the penile glands are parameters for determining the progression of puberty in male rats, and daily exposure to the herbicide metolachlor significantly anticipated the onset of puberty. In relation to age, the MT5 and MT50 groups presented precocious puberty as compared to the control group (Kruskall-Wallis, *P* < 0.05, Figure 5(a)). In relation to body weight at puberty, the MT5 and MT50 groups were lighter than the control group in this phase of sexual development (one-way ANOVA, *P* < 0.05, Figure 5(b)). Note that the weights differed among groups only because it corresponded to an age at puberty in about 4 days earlier than in control animals.

### 3.5. Prepubertal Exposure to Metolachlor Does Not Alter the Growth of the Animals

The assessment of daily weight from weaning until puberty was performed to ascertain possible compromised development caused by the herbicide. The body weight at 23 days old was used as cofactor for correcting the initial body weight of animals, and there was no significant difference among the groups undergoing different treatment regimes with metolachlor. Only the expected variation pertaining to age was observed, showing that body evolution was not affected by the concentrations used (MANCOVA, *P* > 0.05).

## 4. Discussion

Metolachlor is a widely used herbicide that might be related to effect of endocrine chemical disruptor since it was known to alter the aromatase activity in culture cells [23]. So, the focus of this study was to verify the toxicological effects on the reproductive parameters of a commercial formulation of metolachlor *in vivo*, using prepubertal male rats as an experimental model. We demonstrated that prepubertal exposure to metolachlor caused changes in the reproductive endocrinology of male rats, with different effects in the two doses used. The MT5 group presented a reduction in the DHT serum concentrations and an increase in the FSH serum concentrations, while the MT50 group showed an increase in the testosterone, estradiol, and FSH serum concentrations. There were also observed a higher amount of fluid in the seminal vesicles, precocious puberty, and changes in morphology of the seminiferous epithelium of treated animals in both groups.

The gonadotropins are produced and released by the stimulation of hypothalamic GnRH, and testosterone exerts negative feedback on LH production [28]. Despite the increased testosterone serum concentrations, there was no effect on serum LH concentrations in animals treated with metolachlor.

The FSH, which stimulates the production of estradiol by Sertoli cells, was elevated in the treatments, as well as serum estradiol concentrations. This increase in estradiol may also be due to the stimulation of aromatase by metolachlor, as observed in culture cells [23], which leads to greater conversion of estradiol from testosterone [29, 30].

The DHT, converted from testosterone, presented a small reduction in its serum concentrations. The androgenic activity is mainly exerted by these two hormones, being that the DHT has greater bioactivity than testosterone.

Spermatogenesis takes place in the germinative epithelium, a process whose main objective is the production of viable gametes that will be transported by a mature and efficient reproductive system. Because sex hormones may influence the spermatogenesis, we evaluated the morphometry of the seminiferous epithelium in histological sections, and metolachlor exposure increased in the height of the germinal epithelium of seminiferous tubules. The epithelium height is an indicator for the process of spermiogenesis [31] and for the hormonal modifications associated with problems in its architecture [32–34]. Thus, the greatest height of the germinative epithelium may be linked to intense activity on the part of the Sertoli cell, stimulated by the greater levels of circulating estradiol and testosterone. Besides the increased epithelium, the tubular lumen was reduced.

The vesicular gland is androgen-dependent tissue and was used as parameters for evaluating the effects of testosterone level variations. The seminal vesicles were lighter and had a greater amount of liquid in both treatments with metolachlor. As the activity of the vesicular glands is dependent on the action of androgens, it is supposed that the gland is subject to the action of greater amounts of circulating androgens.

The age at puberty is an important component of reproductive development and is influenced by sex hormones, which can determine its anticipation or delay. In rats, the age at puberty onset can be evaluated by the separation of the preputial skin from the glans of the penis [Balanopreputial separation (PPS)] [27, 35] and is triggered by the rise of serum testosterone concentrations in the prepubertal period [27]. Metolachlor exposure caused an anticipation of the puberty, possibly accomplished for the highest testosterone levels (because testosterone was increased in the end of the experimental period), which may have contributed to the change in the age at puberty onset. Because the rats reached puberty at an earlier age, the animals had lower body weights at puberty. However, the animal body weights throughout the experimental period revealed no differences between groups, suggesting that the effects of prepubertal administration of metolachlor are restricted to reproductive development and function.

This study shows, for the first time, the effects on the reproductive development of male treated with metolachlor in the prepubertal period. We conclude that the exposure promotes endocrine problems in reproductive parameters and these changes are reflected by altering the serum concentrations of testosterone, DHT, estradiol, and FSH as well as by causing morphological alterations in androgen-targeted tissues, since the lowest dose used, which may indicate the potential risk of occupational exposure to this herbicide.

## Figures and Tables

**Table 1:** Tubular diameter (*μ*m), epithelial height (*μ*m) and tubular lumen (*μ*m) of seminiferous epithelium in control and treated male rats.

Groups	Tubular diameter (*μ*m)	Epithelial height (*μ*m)	Luminal diameter (*μ*m)
Control	222.9 ± 4.9^a^	46.2 ± 0.8^a,c^	115.7 ± 2.6^a^
MT5	230.6 ± 4.0	53.7 ± 1.8^d^	106.9 ± 2.9^b^
MT50	234.2 ± 3.0^b^	50.3 ± 0.5^b^	111.5 ± 7.2

Doses: control = 0 mg/kg, MT5 = 5 mg/kg and MT50 = 50 mg/kg. Results are expressed as mean ± SEM, ANOVA, *n* = 10 animals/group, ^a/b^differ *P* < 0.05; ^c/d^differ *P* < 0.01.
